# Identification and Functional Investigation of *SOX4* as a Novel Gene Underpinning Familial Atrial Fibrillation

**DOI:** 10.3390/diagnostics14212376

**Published:** 2024-10-25

**Authors:** Wei-Feng Jiang, Yu-Min Sun, Xing-Biao Qiu, Shao-Hui Wu, Yuan-Yuan Ding, Ning Li, Chen-Xi Yang, Ying-Jia Xu, Ting-Bo Jiang, Yi-Qing Yang

**Affiliations:** 1 Department of Cardiology, The First Affiliated Hospital of Soochow University, Suzhou 215006, China; wf.jiang@hotmail.com; 2 Department of Cardiology, Shanghai Jing’an District Central Hospital, Fudan University, Shanghai 200040, China; sunyumin@fudan.edu.cn; 3 Department of Cardiology, Shanghai Chest Hospital, Shanghai Jiao Tong University School of Medicine, Shanghai 200030, China; qiuxingbiao@hotmail.com (X.-B.Q.); wushaohui18@163.com (S.-H.W.); 4 Shanghai Health Development Research Center, and Shanghai Medical Information Center, Shanghai 200031, China; dingyuanyuan@shdrc.org; 5 Department of Cardiology, Putuo Hospital, Shanghai University of Traditional Chinese Medicine, Shanghai 200062, China; frankleelx@163.com; 6 Department of Cardiology, Shanghai Fifth People′s Hospital, Fudan University, Shanghai 200240, China; 13472626672@163.com (C.-X.Y.); xuyingjia@5thhospital.com (Y.-J.X.); 7 Center for Complex Cardiac Arrhythmias of Minhang District, Shanghai Fifth People′s Hospital, Fudan University, Shanghai 200240, China; 8 Department of Cardiovascular Research Laboratory, Shanghai Fifth People′s Hospital, Fudan University, Shanghai 200240, China; 9 Department of Central Laboratory, Shanghai Fifth People′s Hospital, Fudan University, Shanghai 200240, China

**Keywords:** atrial fibrillation, human genetics, sequencing examination, SOX4, biochemical assay

## Abstract

**Background:** Atrial fibrillation (AF) signifies the most prevalent supraventricular arrhythmia in humans and may lead to cerebral stroke, cardiac failure, and even premature demise. Aggregating strong evidence points to genetic components as a cornerstone in the etiopathogenesis of familial AF. However, the genetic determinants for AF in most patients remain elusive. **Methods:** A 4-generation pedigree with idiopathic AF and another cohort of 196 unrelated patients with idiopathic AF as well as 278 unrelated healthy volunteers were recruited from the Chinese population of Han ethnicity. A family-based whole-exome sequencing examination followed by a Sanger sequencing assay in all research subjects was implemented. The functional impacts of the identified *SOX4* mutations were explored via a dual-reporter assay. **Results:** Two new heterozygous *SOX4* mutations, NM_003107.3: c.211C>T; p.(Gln71*) and NM_003107.3: c.290G>A; p.(Trp97*), were observed in the family and 1 of 196 patients with idiopathic AF, respectively. The two mutations were absent in the 278 control individuals. The biochemical measurements revealed that both Gln71*- and Trp97*-mutant SOX4 failed to transactivate *GJA1 (Cx43)*. Moreover, the two mutations nullified the synergistic activation of *SCN5A* by SOX4 and TBX5. **Conclusions:** The findings first indicate *SOX4* as a gene predisposing to AF, providing a novel target for antenatal genetic screening, individualized prophylaxis, and precision treatment of AF.

## 1. Introduction

Atrial fibrillation (AF) represents the most prevalent form of cardiac dysrhythmia in the settings of clinical practices, occurring in up to 1% of the general population globally [1,2]. The incidence of this complex heart rhythm disorder increases drastically with increasing age, rising from roughly 1% in cases under 60 years of age to about 9% in those aged over 65 years, and up to 12% in those 75 years old and above [3]. According to the estimated global burden of disease, AF affects at least 43 million people around the globe [4], and merely in the United States, AF was gauged to affect 5.2 million in the year 2010, with an anticipation of up to 12.1 million in the year 2030 [1]. The overall lifetime risk for developing AF is ~15% in Chinese individuals, ~20% in African American persons, and ~30–40% in White folks [1]. Considering that up to two-thirds of subjects with silent/subclinical AF are symptomless and probably undiagnosed, the true incidence of AF is evidently underreckoned [5,6,7]. It is currently estimated that the actual prevalence of AF is at least 3–4% when asymptomatic AF is included [8]. AF is a major contributor to an extensive variety of adverse clinical consequences, including degraded health-associated quality of life with impaired physical ability [9,10,11,12,13], thromboembolic/ischemic cerebral stroke [14,15,16,17,18], early-onset cognitive decline/dementia [19,20,21,22,23], chronic kidney disease/acute renal injury [24,25,26], myocardial infarction [27,28,29,30], congestive cardiac failure [31,32,33], life-threatening ventricular dysrhythmias [34,35,36,37], and even cardiovascular demise [38,39,40,41]. Rather, AF confers an increased risk of multiple adverse outcomes, encompassing a 6-fold risk of chronic renal disease, 1.5-fold risk of dementia/intellectual impairment, 2.4-fold risk of stroke, 1.5-fold risk of myocardial infarction, 1.3-fold risk of peripheral artery disease, 5-fold risk of cardiac failure, and 2-fold risk of cardiac lethality [1]. In retrospective clinical research of Medicare beneficiaries over 65 years of age, after the diagnosis of incident AF, the most common outcome during the first 5 years was mortality (48.8%); the next most frequent was heart failure (13.7%), followed by new-attack cerebral stroke (7.1%), hemorrhage of the gastrointestinal tract (5.7%), and myocardial infarction (3.9%) [1,42]. Consequently, AF has led to substantive morbidity and considerable mortality along with enormous economic incumbrance [1,43,44,45,46,47]. Despite the pronounced significance, the pathogenic mechanisms underlying the initiation and maintenance of AF remain incompletely understood.

It is well known that the etiological mechanisms initiating and sustaining AF are exceedingly complex and extremely distinct, and both non-inherited/environmental hazard factors and inheritable defective ingredients may result in the occurrence and sustainment of AF [1,2,3,48,49,50,51]. There is an increasing amount of non-genetic factors predicted to enhance the susceptibility to AF, encompassing advancing age, alcohol consumption, smoking, inhalation of airborne particulate substances, sedentary lifestyles, obesity, primary arterial hypertension, obstructive sleep apnea, rheumatic heart malady, acute myocardial infarction, dilated cardiomyopathy, peri-atrial inflammation/pericarditis/myocarditis, pneumonia/pulmonary embolism, cardiothoracic surgery, diabetes mellitus, hyperthyroidism, metabolic disorder, β-Thalassemia, and cardiac autonomic nerve imbalance [1,3,48,49,50,52,53,54,55,56,57,58,59,60]. However, accumulating epidemiological evidence has convincingly indicated that heritable causative components exert pivotal roles in the development and maintenance of AF, especially for familial AF [2,49,50,51]. To date, >60 genes causative for AF have been discovered, of which the vast majority code for ion channel subunits of potassium (such as KCNQ1), natrum (such as SCN5A) and calcium (such as CACNA1A), gap junction proteins/connexins (such as GJA1/Cx43), cardiac transcription factors (such as TBX5), signaling molecules (such as ANP), and myocardial structural proteins (such as TTN) [2,49,50,51,61,62,63,64,65,66,67,68,69,70,71,72]. Additionally, genome-wide genetic association investigations have unveiled that genetic variants at over 135 chromosomal loci are associated with an increased vulnerability to AF. However, the functionally pathogenic effects of most of these AF-associated variants remain to be validated experimentally [2,73]. Nevertheless, due to conspicuous genetic heterogeneity, the heritable determinants responsible for AF in most patients remain to be ascertained. In addition, there exist potential confounders in genetic studies, such as population stratification and environmental factors, which could affect the interpretation of the results. To address these gaps, the scope of the current research was to identify a novel gene responsible for AF and unravel its functional characteristics.

## 2. Materials and Methods

### 2.1. Enrollment and Clinical Study of Individuals

The present retrospective clinical investigation was fulfilled strictly in harmony with the ethical tenets summarized in the Declaration of Helsinki. The institutional ethical panel from Shanghai Chest Hospital approved the protocols applied to the human investigation (with an approved protocol code of KS1101 and an ethical approval date of 12 April 2011 for the study protocol). The research participants or their legal guardians provided signed informed consent forms at initial recruitment, before the commencement of clinical studies.

For the current human investigation, a 4-generation pedigree with a high incidence of idiopathic AF (arbitrarily termed Family AF-01) and another cohort of 196 unrelated patients with idiopathic AF as well as 278 unrelated healthy volunteers without a familial history of AF were recruited from the Chinese population of Han ethnicity between March 2012 and September 2024. The inclusion criteria for the family members and another cohort of AF patients were informed consent and no known causes for AF. The exclusion criteria for the family members and another cohort of AF patients encompassed no informed consent and known causes for AF. Individual-level phenotypic data, which comprised surveys of personal, familial, and medical histories, medical records, transthoracic echocardiographic images, 12-lead electrocardiograms, physical examination findings, and routine bioassay reports, were collected from each research participant in the clinical study. A 24-h or 48-h Holter/ambulatory electrocardiographic monitor was performed whenever indicated. Clinical diagnosis and categorization of AF were implemented as elaborated elsewhere [1]. A whole venous blood sample (3.0 mL) was collected from each study participant and utilized to routinely isolate genomic DNA from the blood leukocytes.

### 2.2. Genetic Examination

Whole-exome sequencing (WES) assays in three AF-affected and two healthy members from Family AF-01 were conducted as previously described [62,68,74,75,76,77]. In brief, a sample of 4 µg of genomic DNA from each family member chosen for the WES assay was used to construct an exome DNA library. Sequencing of exome DNA libraries was completed with the HiSeq Sequencing Kit (Illumina, San Diego, CA, USA) on the HiSeq4000 Genome Analyzer (Illumina, San Diego, CA, USA) as per the manufacturer’s manuals. Raw exome-sequencing data were subject to a procedure using Pipeline version 1.9 (Illumina, San Diego, CA, USA) to read bases and align to the human genome (referential GRCh37/hg19) using BWA (version 0.7.12). Calling of sequence variations was implemented by employing SAMtools (version 1.9) and GATK (version 4.1.8), followed by annotation and filtering of genetic variants with ANNOVAR (an online version at http://www.openbioinformatics.org/annovar/) as previously described [62,68,74,75,76,77]. Briefly, based on the possible inheritance patterns of AF in the studied pedigree (Family AF-01), the genetic variations that did not match any reasonable inheritance fashion of AF were filtered out. Then the population genetics databases of gnomAD and dbSNP were applied to filter out the synonymous polymorphic variations and any genetic variation with a frequency of >0.001 in either of the two databases. The non-synonymous variations (encompassing nonsense, missense, readthrough, and small insertion/deletion variations as well as splicing donor/acceptor variations) with minor allele frequencies of <0.001 that passed the pedigree assay with reasonable inheritance modes of AF were annotated with ANNOVAR. The deleterious variants annotated by ANNOVAR were selected as candidate pathogenic variants to be subject to validation by Sanger sequencing analysis in the entire family (Family AF-01). Once a rare deleterious variation was confirmed to co-segregate with AF in the whole pedigree, hence a potentially AF-causative mutation, a Sanger sequencing assay of the coding exons along with flanking introns of the genes containing the identified AF-causing mutation was conducted in Family AF-01, and another cohort of 196 unrelated patients with idiopathic AF as well as the 278 healthy subjects served as controls. Additionally, such publicly accessible population genetics databases as dbSNP and gnomAD were retrieved as previously described [74] to check the novelty of the discovered AF-causing mutations.

### 2.3. Construction of Gene-Expressing Plasmids

Given that *SOX4* is an intronless gene, a 1496-bp fragment including the entire coding exon of wild-type human *SOX4* (Nucleotide accession version: NM_003107.3/GenBank accession version: NC_000006.12) was amplified from human genomic DNA via polymerase chain reaction (PCR) utilizing the DreamTaq™ DNA Polymerase Kit (Thermo Scientific, Carlsbad, CA, USA) with a *SOX4*-specific pair of oligonucleotide primers of 5′-GTAGAATTCCGCGAGGGTGTGAGCGCGC-3′ (forward) and 5′-GTAGTCGACGGCCCTTCTCCCTGCCTGC-3′ (backward). The amplicons containing a whole coding exon of wild-type human *SOX4* and the pCI mammalian expression vector (Promega, Madison, WI, USA) were doubly cut by *EcoR*I and *Sal*I, isolated by gel electrophoresis, purified with the GeneJET™ Gel Extraction Kit (Thermo Scientific, Carlsbad, CA, USA), and ligated using T_4_ DNA ligase to generate the wild-type human SOX4-pCI recombinant vector. With the wild-type human SOX4-pCI vector utilized as a template, the Gln71*-mutant human SOX4-pCI vector was procured by site-targeted mutagenesis with the Phusion™ Site-Directed Mutagenesis Kit (Thermo Scientific, Carlsbad, CA, USA) in addition to a complimentary pair of oligonucleotide primers (forward: 5′-TTCATGGTGTGGTCGTAGATCGAGCGGCGCA-3′; backward: 5′-TGCGCCGCTCGATCTACGACCACACCATGAA-3′). Similarly, the Trp97*-mutant human SOX4-pCI vector was yielded with a complimentary pair of primers of 5′-CTGGGCAAACGCTAGAAGCTGCTCAAAGA-3′ (forward) and 5′-TCTTTGAGCAGCTTCTAGCGTTTGCCCAG-3′ (backward). Additionally, a 1904-bp promoter fragment (from −97 to −2000 upstream of the transcriptional initial site, with the first transcribed nucleotide being numbered as +1) of the human *GJA1/Cx43* gene (GenBank accession version: NC_000006.12) was amplified from a template of human genomic DNA by PCR utilizing the DreamTaq™ DNA Polymerase Kit (Thermo Scientific, USA) and a *GJA1*-specific pair of oligonucleotide primers of 5′-GCAGAGCTCGACCTAACAAAACATAACAG-3′ (forward) and 5′-GCACTCGAGGAGGTAGGGAGGAGGCTGG-3′ (backward). The amplified *GJA1* promoter and the vector of pGL3-Basic (Promega, Madison, WI, USA) were doubly digested by *Sac*I and *Xho*I, isolated by gel electrophoresis, extracted with a gel extraction kit, and ligated with T_4_ DNA ligase to construct the GJA1-luciferase (GJA1-luc) reporter vector expressing firefly luciferase. Similarly, a 2220-bp promoter fragment (from −751 to −2970 upstream of the transcriptional initiation site) of the human *SCN5A* gene (GenBank accession version: NC_000003.12) was amplified utilizing a *SCN5A*-specific pair of oligonucleotide primers of 5′-CTGGGTACCTGAACACTTAGGATTTGTGC-3′ (forward) and 5′-CTGCTCGAGTACACATCTACCTGAACAC-3′ (backward), doubly cut with *Kpn*I and *Xho*I, and inserted into the pGL3-Basic vector (Promega, USA) at the *Kpn*I-*Xho*I sites to construct the SCN5A-luc reporter vector expressing firefly luciferase. The TBX5-pcDNA3.1 plasmid expressing wild-type human TBX5 was produced as previously described [78]. The integrity of all the recombinant expression vectors was validated by DNA sequencing examination.

### 2.4. Cellular Transfection and Dual-Reporter Gene Measurement

COS-7 and HEK293 cells were routinely cultivated as described elsewhere [79,80]. Cells were counted with an automated hemocytometer (Thermo Scientific, Waltham, MA, USA) and seeded in a 24-well plate (Thermo Scientific, Rochester, NY, USA), maintained for 24 h, and then transiently transfected with various expressing plasmids utilizing the TurboFect Transfection Reagent (Thermo Scientific, Carlsbad, CA, USA). Specifically, COS-7 cells were transfected with 3 ng of pGL4.75 (Promega, Madison, WI, USA), 1.6 μg of GJA1-luc, and 0.4 μg of each recombinant expression plasmid (empty pCI, wild-type human SOX4-pCI, Gln71*-mutant human SOX4-pCI, or Trp97*-mutant human SOX4-pCI, singly or together). For the synergistic activation analysis, HEK293 cells were transfected with 1 ng of pGL4.75 (Promega, Madison, WI, USA), 0.8 μg of SCN5A-luc, and 0.4 μg of each recombinant expression plasmid (empty pCI, wild-type human TBX5-pcDNA3.1, wild-type human SOX4-pCI, Gln71*-mutant human SOX4-pCI, or Trp97*-mutant human SOX4-pCI, alone or in combination). Here, as an internal control, the plasmid of pGL4.75 (Promega, Madison, WI, USA) expressing renilla luciferase was applied to balance transfection efficiency. The plasmid of empty pCI (Promega, Madison, WI, USA) was employed as an external negative control. Cells were harvested 36 h post cellular transfection, lysed, and the firefly/renilla luciferase activities in the cellular lysates were measured on a luminometer (the GloMax^®^ Discover System; Promega, Madison, WI, USA) with the Dual-Glo^®^ Luciferase Assay System (Promega, Madison, WI, USA) as per the manuals and analyzed as described previously [81,82,83]. Briefly, the promoter activities were expressed as the ratios of firefly to renilla luminescence intensities. For every recombinant expression plasmid, at least three independent cellular transfection tests in vitro were implemented in triplicates, and the mean of three independent experimental results was used for comparison between or among groups.

### 2.5. Statistical Analysis

The statistical assay was conducted with the aid of SPSS version 17.0 (SPSS, Chicago, IL, USA) as described previously [84,85,86]. Briefly, such quantitative variables as ages and promoter activities were expressed as means ± standard deviations ($\bar{x}\;$± SD). Such qualitative variables as sex and family history of AF were expressed as a frequency numeral (%). Unpaired Student’s *t*-test was used to compare continuous parameters between the two groups. A one-way analysis of variance (ANOVA) along with the Tukey–Kramer post-hoc test was applied to the comparison of quantitative variables among three or more groups. A Chi-square or Fisher’s exact test was utilized to compare categorical parameters between the two groups. A two-tailed *p* < 0.05 indicated a statistical difference.

## 3. Results

### 3.1. Baseline Demographic and Clinical Profiles of the Pedigree with AF and Other Study Participants

**Figure 1:**
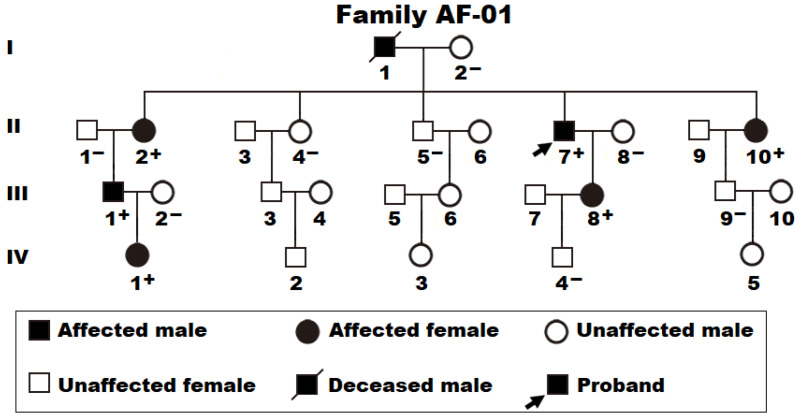
Pedigree suffering from atrial fibrillation. The pedigree was arbitrarily termed as Family AF-01. “+” signifies a member harboring the discovered heterogeneous *SOX4* mutation; “–” represents a non-carrier of the discovered *SOX4* mutation.

As shown clearly in Figure 1, a 27-member family spanning 4 generations with a high incidence of AF (arbitrarily termed Family AF-01) was enrolled, encompassing 26 living family members.

**Figure 2:**
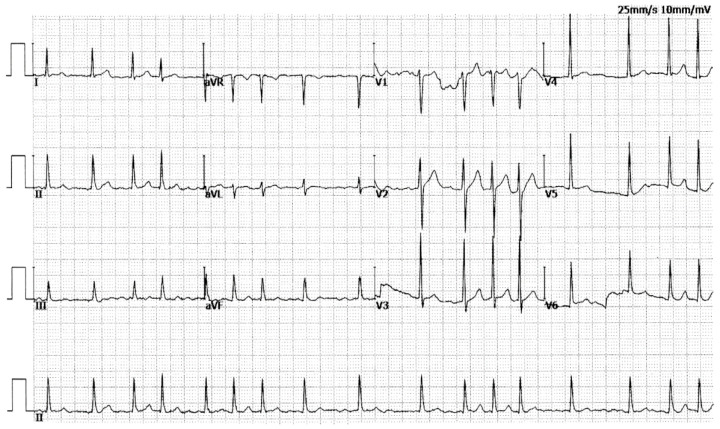
A representative electrocardiogram recorded from the proband (II-7) of Family AF-01. The standard 12-lead electrocardiogram illustrates atrial fibrillation.

In Family AF-01, seven members, including three male members and four female members, were diagnosed with AF according to the electrocardiographic findings. No well-established nonheritable factors predisposing to AF were found in the family members, such as advancing age, obstructive sleep apnea, coronary heart disease, cardiac surgery, dilated cardiomyopathy, pulmonary heart disease, myocarditis, heart failure, primary hypertension, diabetes mellitus, hyperthyroidism, and chronic kidney disease. The proband (member II-7 in Family AF-01), a fifty-seven-year-old male member with twelve years of AF history, was admitted to the hospital because of recurrent syncope. He had an electrocardiogram-documented AF as shown in Figure 2 and then underwent a successful termination of AF by catheter-based radiofrequency ablation.

The proband’s elder sister (member II-2 in Family AF-01), a sixty-five-year-old female individual with seventeen years of AF history, experienced a successful termination of AF by radiofrequency ablation when she was 52 years old. The proband’s father (member I-1 in Family AF-01) had thirty-six years of AF history and died of a thromboembolic cerebral stroke at 71 years of age. The index patient’s other relatives with AF possessed a definite history of taking antiarrhythmic drugs, but none of them experienced surgical or interventional therapy for AF at the time of recruitment. The proband’s unaffected relatives (ten male and ten female subjects) denied a history of AF occurrence, with their electrocardiograms being normal. The basic demographic and clinical characteristic data of the family members with different types of AF are summarized in Table 1.

In addition, all the available family members affected by AF also had dental developmental abnormalities and hypoplastic fifth distal phalanges/nails, as well as mild facial dysmorphisms characteristic of micrognathia with a bulbous nasal tip.

In addition, another cohort of 196 unrelated patients with idiopathic AF and a total of 278 unrelated healthy volunteers without a familial history of AF were enrolled from the Chinese population of Han ethnicity and clinically evaluated. The baseline demographic and clinical characteristic profiles of the cohort of AF patients and the healthy volunteers employed as controls are provided in Table 2.

### 3.2. Discovery of Two New AF-Causative Mutations in SOX4

The WES assay was fulfilled in three AF-affected members (II-7, II-10, and III-8) and two healthy members (III-9 and IV-4) from Family AF-01 (Figure 1), which yielded about 23 Gb of DNA sequences for each member, with approximately 96% mapped to the human reference genome (referential GRCh37/hg19) and about 74% aligned with the target sequences. An average of 17,094 non-synonymous genetic variations per member (ranging from 16,102 to 18,150) passed pedigree filtering based on the presumable transmission modes, among which 13 heterozygous missense/nonsense variations with a minor allele frequency of <0.1% went through filtering by ANNOVAR and were shared by all the three AF-inflicted members (II-7, II-10, and III-8 from Family AF-01) who were subject to WES, as provided in Table 3.

Sanger sequencing examination in all the pedigree members as indicated in Family AF-01 revealed that only the nonsense variation of chr6: 21,594,976C>T (GRCh37.p13/hg19: NC_000006.11), equivalent to chr6: 21,594,745C>T (GRCh38.p14/hg38: NC_000006.12) or NM_003107.3: c.211C>T; p.(Gln71*) in *SOX4*, was verified to be in co-segregation with AF in the whole pedigree. Furthermore, a Sanger sequencing assay of the complete coding region of the *SOX4* gene was executed in all the study subjects using the oligonucleotide primers given in Table 4, which uncovered that the mutation of NM_003107.3: c.211C>T; p.(Gln71*) in *SOX4* existed in all the AF-affected family members but in none of the healthy family members as well as the 278 unrelated control subjects. Genetic assay of Family AF-01 suggested that AF was inherited in an autosomal-dominant fashion.

**Figure 3:**
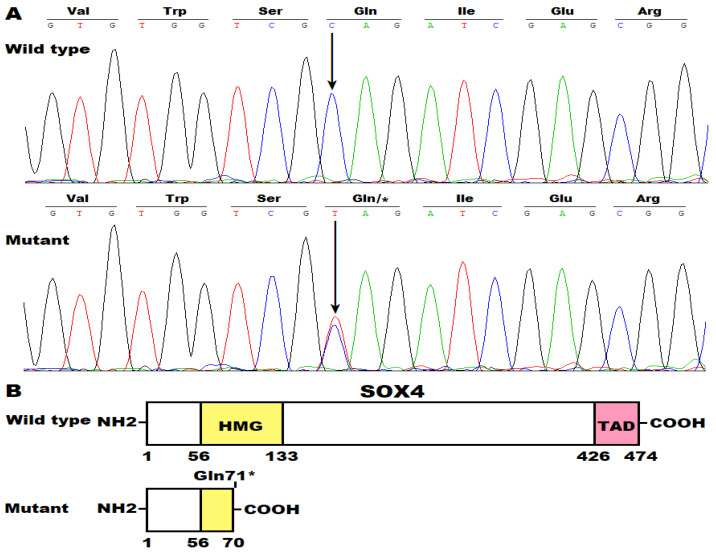
A novel *SOX4* mutation predisposing to atrial fibrillation. (**A**) Sequencing electropherograms exhibiting the heterogeneous SOX4 mutation identified in the index patient with atrial fibrillation (mutant) along with its wild-type control detected in an unaffected subject (wild type). An arrow pinpoints where the mutation occurs. (**B**) Schematic drawings describing the main structural domains of SOX4. TAD: transcriptional activation domain; HMG: high mobility group.

The sequencing chromatograms showing the heterozygous c.211C>T mutation in *SOX4* together with its wild-type control are presented in Figure 3A. The schemas delineating the main structural domains of the wild-type and Gln71*-mutant human SOX4 proteins are provided in Figure 3B.

**Figure 4:**
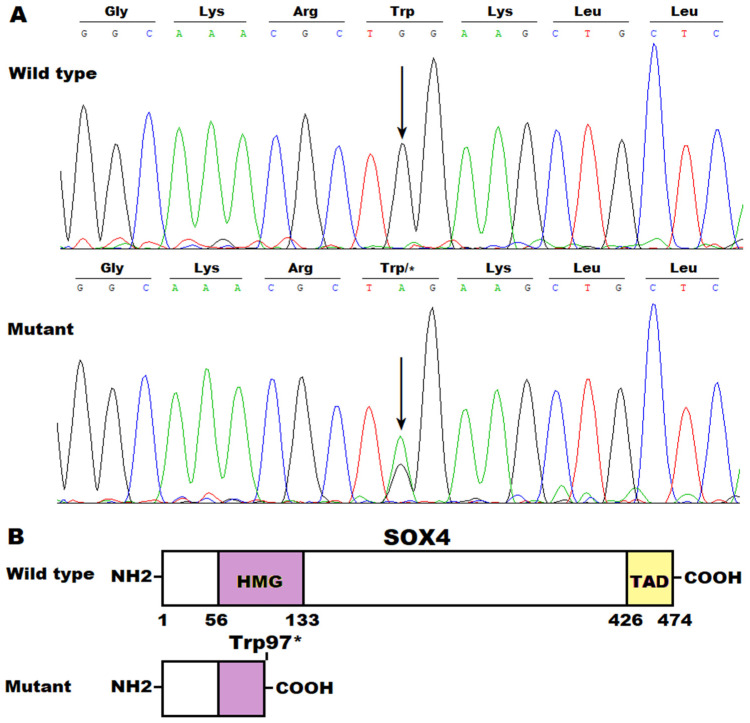
A novel SOX4 mutation contributing to atrial fibrillation. (**A**) Sequencing electropherograms displaying the heterogeneous SOX4 mutation detected in one of the cohort patients with atrial fibrillation (mutant) and its wild-type control detected in a control individual (wild type). An arrow directs where the mutation occurs. (**B**) Schematic diagrams illustrating the main structural domains of SOX4. HMG: high mobility group; TAD: transcriptional activation domain.

Furthermore, a Sanger sequencing assay of the whole coding region of the SOX4 gene was also conducted in another cohort of 196 unrelated patients with idiopathic AF, which unveiled a heterozygous SOX4 mutation of NM_003107.3: c.290G>A; p.(Trp97*) in one female patient aged 47 years with no family history of AF, who also had delayed intellectual development, bilateral 5th finger clinodactyly, and dysplastic 5th toenails, as well as mild facial dysmorphisms characteristic of upturned nares and a wide mouth with cupid bow. Echocardiographic images demonstrated her normal ventricular function and an enlarged left atrium (48 mm for the left atrial diameter). This mutation was neither found in her parents (indicating a de novo mutation) nor observed in the 278 control subjects. The sequencing chromatograms showing the heterozygous c.290G>A mutation in SOX4 along with its wild-type control are presented in Figure 4A. The schemas describing the main structural domains of the wild-type and Trp97*-mutant human SOX4 proteins are provided in Figure 4B.

**Figure 5:**
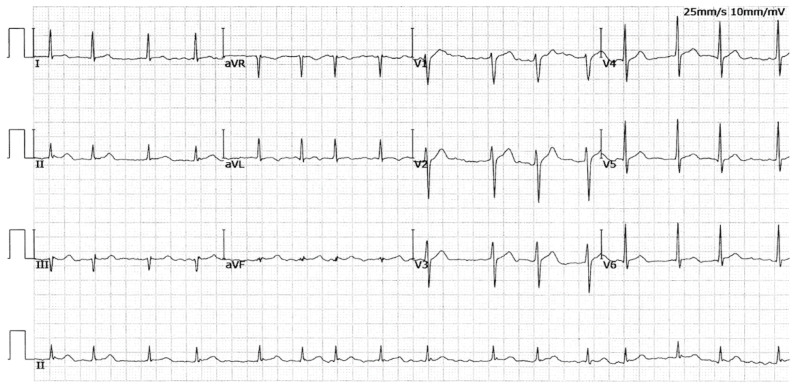
A representative electrocardiogram from the patient harboring the SOX4 c.290G>A mutation. The electrocardiogram documents atrial fibrillation.

A representative electrocardiogram showing AF from the AF patient carrying the SOX4 c.290G>A mutation was given in Figure 5.

Neither of the detected two SOX4 nutations accountable for AF was released from the databases of gnomAD and dbSNP, indicating their novelty.

### 3.3. Inability of Gln71*- or Trp97*-Mutant SOX4 to Transcriptionally Activate GJA1

**Figure 6:**
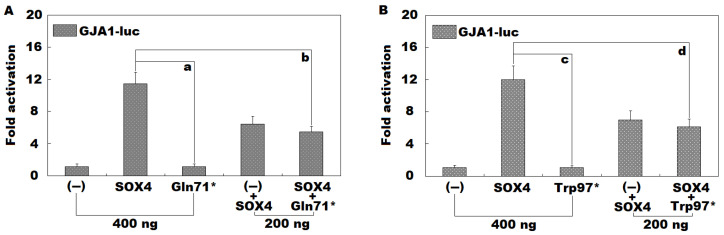
Functional failure of Gln71*- or Trp97*-mutant SOX4. In COS-7 cells cultivated in vitro, dual-reporter measurement of the transactivation of the *GJA1/Cx43* promoter-driven firefly luciferase by wild-type SOX4 or Gln71*-mutant SOX4 (Gln71*) or Trp97*-mutant SOX4 (Trp97*), separately or in combination, unveiled that the Gln71* mutant (**A**) or Trp97* mutant (**B**) failed to transactivate *GJA1/Cx43*. Here “a” and “c” mark *p* < 0.001, and “b” and “d” indicate *p* < 0.01, when compared with wild-type SOX4 (400 ng).

As given in Figure 6A, in maintained COS-7 cells expressing multiple vectors, including empty pCI as an external negative control (−), wild-type human SOX4-pCI (SOX4), and Gln71*-mutant human SOX4-pCI (Gln71*), singly or in combination, SOX4 (simulating the physiological state of a healthy person) and Gln71* (simulating the pathological condition of an AF patient with a homozygous SOX4 mutation of Gln71*) transactivated the *GJA1* promoter by ~11-fold and ~1-fold, respectively (SOX4 versus Gln71*: t = 12.4897; *p* = 0.0002). When SOX4 and Gln71* were co-transfected (simulating the pathological condition of an AF patient with a heterozygous SOX4 mutation of Gln71*), the induced transcriptional activity on the *GJA1* promoter was ~5-fold (SOX4 versus Gln71* + SOX4: t = 6.6464; *p* = 0.0027). Moreover, equivalent statistical results were generated when multiple comparison analyses were conducted (F = 76.607, *p* = 1.842 × 10^−7^). Specifically, for SOX4 versus Gln71*, t = 10.3407; *p* < 0.0001; for SOX4 versus Gln71* + SOX4, t = 6.0200; *p* < 0.0001; for SOX4 + (−) versus Gln71* + SOX4, t = 0.9867; *p* = 0.6296. Similarly, as exhibited in Figure 6B, SOX4 (simulating the physiological state of a healthy person) and Trp97* (simulating the pathological condition of an AF patient with a homozygous SOX4 mutation of Trp97*) transactivated the *GJA1* promoter by ~12-fold and ~1-fold, respectively (SOX4 versus Trp97*: t = 10.8912; *p* = 0.0004). When SOX4 and Trp97* were co-transfected (simulating the pathological condition of an AF patient with a heterozygous *SOX4* mutation of Trp97*), the induced transcriptional activity on the *GJA1* promoter was ~6-fold (SOX4 versus Trp97* + SOX4: t = 5.1208; *p* = 0.0069). Moreover, equivalent statistical results were given when multiple comparison analyses were carried out (F = 58.717, *p* = 6.583 × 10^−7^). Specifically, for SOX4 versus Trp97*, t = 10.9200; *p* < 0.0001; for SOX4 versus Trp97* + SOX4, t = 5.8667; *p* = 0.0003; for SOX4 + (−) versus Trp97* + SOX4, t = 0.8733; *p* = 0.8354.

### 3.4. Failure of Gln71*- or Trp97*-Mutant SOX4 to Transactivate SCN5A Alone or in Synergy with TBX5

**Figure 7:**
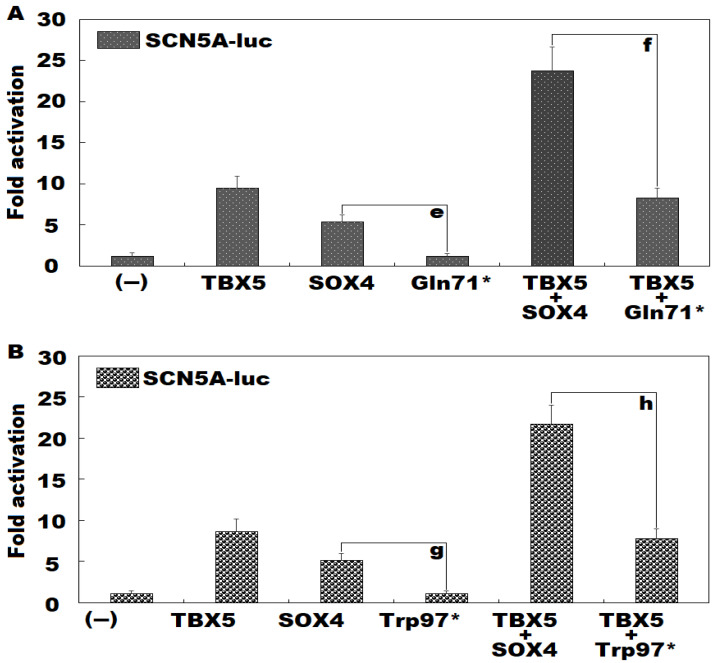
No Synergistic Transactivation of *SCN5A* Between TBX5 and Gln71*- or Trp97*-Mutant SOX4. In cultured HEK293 cells, dual-reporter gene assessment of the synergistic transactivation of the *SCN5A* promoter by TBX5 and SOX4 demonstrated that the synergy was disrupted by the Gln71* (**A**) or Trp97* (**B**) mutation. Herein “e”, “f” and “g” denote *p* < 0.005, and “h” means *p* < 0.001 when compared with the corresponding wild-type counterparts.

As manifested in Figure 7A, in cultivated HEK-293 cells expressing multiple plasmids, encompassing empty pCI (−), wild-type human SOX4-pCI (SOX4), and Gln71*-mutant human SOX4-pCI (Gln71*), separately or both, SOX4 and Gln71* transactivated the *SCN5A* promoter by ~5-fold and ~1-fold, respectively (SOX4 versus Gln71*: t = 8.3656; *p* = 0.0011). In the existence of TBX5, SOX4 and Gln71* transactivated the *SCN5A* promoter by ~24-fold and ~8-fold, respectively (SOX4 + TBX5 versus Gln71* + TBX5: t = 8.4538; *p* = 0.0011). Besides, similar statistical results were produced when multiple comparisons were performed (F = 1047.220, *p* = 3.092 × 10^−9^). Specifically, for SOX4 versus Gln71*, t = 4.2333; *p* = 0.0394; for TBX5 + SOX4 versus TBX5 + Gln71*, t = 15.4567; *p* < 0.0001. Similarly, as shown in Figure 7B, SOX4 and Trp97* transactivated the *SCN5A* promoter by ~5-fold and ~1-fold, respectively (SOX4 versus Trp97*: t = 7.3407; *p* = 0.0018). In the existence of TBX5, SOX4 and Trp97* transactivated the *SCN5A* promoter by ~22-fold and ~8-fold, respectively (SOX4 + TBX5 versus Trp97* + TBX5: t = 9.3766; *p* = 0.0007). Moreover, similar statistical results were obtained when multiple comparison analyses were conducted (F = 104.290, *p* = 1.845 × 10^−9^). Specifically, for SOX4 versus Trp97*, t = 3.9567; *p* = 0.0259; for SOX4 + TBX5 versus Trp97* + TBX5, t = 13.9267; *p* < 0.0001.

**Figure 8:**
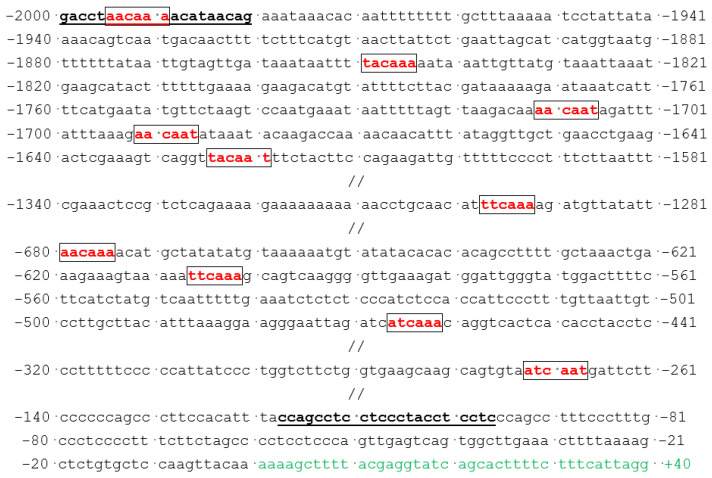
Multiple SOX4-binding sites located within the promoter of *GJA1/Cx43*. Red color was used to mark the SOX4-binding sites; green color: the first exon mRNA; underlined bases in bold: primer sequences.

In addition to multiple SOX4-binding target sites, the congruous DNA sequences of the 5′-WWCAAW-3′ (5′-A/TA/TCAAA/T-3′) motifs within the promoter of *GJA1/Cx43* (GenBank accession version: NC_000006.12) were located and exhibited in Figure 8.

**Figure 9:**
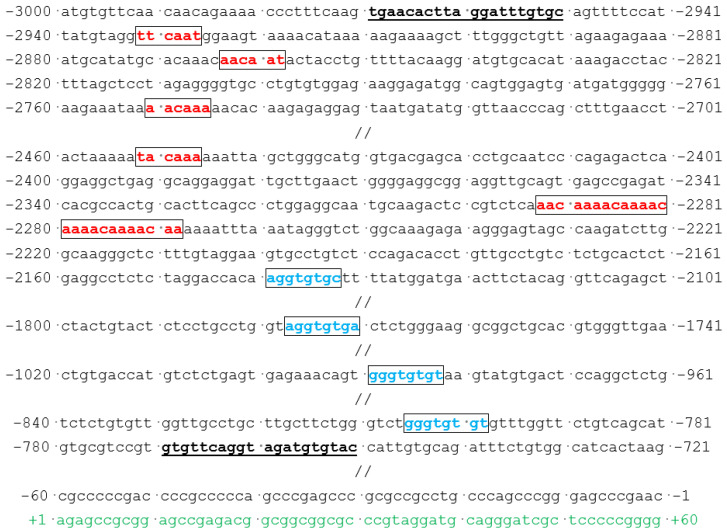
Multiple SOX4- and TBX5-binding sites mapped within the promoter of *SCN5A*. Red color was used to mark the SOX4-binding sites; blue color: the TBX5-binding sites; green color: the first exon mRNA; underlined bases in bold: primer sequences.

Multiple SOX4- and TBX5-binding sites in the promoter of *SCN5A* (GenBank accession version: NC_000003.12) were located and displayed in Figure 9.

## 4. Discussion

The SOX family of transcription factor proteins is characteristic of a DNA-binding domain with high homology to the so-called high-mobility group (HMG) box of the sex-determining region Y (*SRY*) gene [87]. To date, there have been 20 members of the SOX family discovered in all vertebrates, which are categorized into 8 groups ranging from SOXA to SOXH [87]. It has been substantiated that the SOX family proteins play critical roles in the development as well as pathological processes of most organs and tissues from the ectoderm, mesoderm, and endoderm, including the heart, central nervous system, pancreas, bone, retina, cartilage, vasculature, hematopoietic system, and lymphatic system, and a large number of genetic diseases caused by genetically compromised SOX proteins, or termed ‘SOXopathies,’ have been reported, which are involved in the cardiovascular system, reproductive system, muscular system, nervous systems, ocular and auditory systems, urinary system, skin and hair, and skeleton [87,88]. SOX4, together with SOX12 and SOX11, comprises the SOXC group, and SOXC proteins, which share almost identical DNA-binding domains and highly conserved transactivation domains, are amply expressed in many types of progenitor cells and exert redundant functions in regulating cellular survival, differentiation, and fatal determination in response to a great variety of signaling pathways [87]. *SOX4*, a single-exon gene mapped on human chromosome 6p22.3, coding for a 474-amino acid protein [89]. The SOX4 protein possesses 2 functionally key structural domains, including an HMG box and a transcriptional activation domain (TAD) at the C-terminus. The HMG box is responsible for DNA binding, bending, subcellular trafficking, and nuclear distribution, while TAD functions to activate the expression of target genes and mediate the interaction with other transcription factors or cofactors [87]. Previous experiments have revealed that SOX4 is abundantly expressed in the cardiovascular system in both mice and humans, playing critical roles in the embryonic development and postnatal remodeling of the hearts and vessels [90,91,92,93,94,95,96]. Noticeably, SOX4 has been validated to transcriptionally regulate the expression of *GJA1*-encoded Cx43, alone or synergistically with the T-box family of transcription factors, including TBX5 [97]. Additionally, TBX5 has been verified to drive the expression of multiple genes, including *SCN5A*, *PITX2*, *GJA1*, and *RyR2* [98,99,100], and mutations in *SCN5A*, *PITX2*, *GJA1*, and *RyR2* have been reported to cause AF [2,49]. In the current human research, the Gln71* and Trp97* mutations in SOX4 uncovered in patients affected with AF were predicted to produce truncated SOX4 proteins losing TAD along with about halves of the HMG domains, hence anticipated to fail to bind target DNA and activate transcription, and quantitative biochemical measurements demonstrated that Gln71*- or Trp97*-mutant SOX4 failed to transactivate the expression of *GJA1* as well as *SCN5A* in synergy with TBX5. These findings indicate that *SOX4* haploinsufficiency contributes to the pathogenesis of AF probably by reducing the expression of such genes as *GJA1* and *SCN5A*.

The key roles of SOX4 in cardiovascular development and remodeling have been demonstrated in animals and humans [90,91,92,93,94,95,96]. In mice, SOX4 is predominantly expressed in the endocardial tissue, including both the outlet tract and atrioventricular canal, and deletion of *Sox4* led to embryonic death because of circulatory failure at embryonic day 14, which was a result of abnormal development of the endocardial ridges into the semilunar valves as well as the outflow tract of the muscular ventricular septum [90,91]. Additionally, in mice with conditional null of *Sox4* alleles (*Sox4*^fl-/fl-^), embryos died from the same cardiovascular developmental defects as observed in the *Sox4*-knockout mice (*Sox4*^−/−^) [92]. Both in mice with acute myocardial infarction and in neonatal murine cardiomyocytes treated with hydrogen peroxide (H_2_O_2_), the expression of SOX4 was markedly increased, and overexpression of SOX4 induced cardiomyocyte apoptosis with or without H_2_O_2_, whereas knockdown of *Sox4* could alleviate H_2_O_2_-induced myocardial apoptosis [93]. Furthermore, silencing *Sox4* diminished the area of cardiac infarction, improved heart function, and reversed *Sox4*-induced cardiomyocyte apoptosis in mice with myocardial infarction [93]. Liu and colleagues [94] performed RNA-sequencing analysis of cardiac tissue specimens from patients with heart failure caused by dilated or hypertrophic cardiomyopathy and healthy donors to determine the RNA expression levels of 20 SOX genes and found that only the *SOX4* and *SOX8* RNA levels were significantly upregulated in the heart failure groups, irrespective of four SOX genes whose RNA expression levels were remarkably increased in the dilated or hypertrophic cardiomyopathy group compared to the healthy donor group [94]. Additionally, a moderate to strong association of the RNA level of *SOX4*/*SOX8* with fibrotic genes was observed, and via a meta-analysis of epigenetics and whole-genome association data, several genomic variants accountable for heart failure were linked to *SOX4* or *SOX8* [94]. Cheng and coworkers [95] identified SOX4 as an atherosclerotic marker in mouse aortic tissues and distinct human arteries (human coronary arteries and human renal arteries), in addition to a cancer marker. Endothelial cell-specific overexpression of SOX4 promoted atherogenesis and increased endothelial-to-mesenchymal transition [95]. In addition, suppression of circHDAC9, a circular RNA, could dramatically lower the level of SOX4 and significantly attenuate myocardial ischemia/reperfusion injury by mediating the miR-671-5p/SOX4 signaling pathway [96]. Importantly, rapidly evolving strong evidence has demonstrated that cardiovascular developmental abnormalities, especially the myocardial sleeves clothing the systemic and pulmonary veins at their junctions with the atria, underly ectopic pace-making electrical activities and are common anatomic matrixes underpinning AF [101,102,103]. Taken collectively, these research results highlight the fundamental roles of SOX4 in proper cardiovascular development and indicate that genetically defective SOX4 predisposes to AF very likely by creating an abnormal electrophysiological substrate in favor of AF.

Previously in humans, pathogenic *SOX4* variants encompassing causative missense and truncating variants were involved in the etiopathogenesis of Coffin–Siris syndrome, a rare genetic disease characterized by multi-systemic developmental anomalies with a phenotypic spectrum varying from mild cognitive delays to intellectual disabilities with various congenital deformities, including craniofacial dysmorphism, hypoplasia or absence of the fifth distal phalanges/nails, and dental abnormalities [87,104]. Furthermore, copy number variants resulting in entire gene deletion or duplication of *SOX4* were associated with skeletal and mesomelic dysplasia as well as cardiac developmental defects but were not causally linked to any neurodevelopmental disorder [87,105,106,107,108]. In the present investigation, two novel *SOX4* mutations were implicated with AF as a notable clinical feature of Coffin–Siris syndrome, thus expanding the *SOX4*-linked phenotypic spectrum. Considering that a significant proportion of AF is paroxysmal or subclinical without symptoms [1], this study indicates that a long-term electrocardiographic screening of the patients with Coffin–Siris syndrome caused by *SOX4* mutations is necessary for the discovery of AF as a component of broader syndromic conditions.

It has been demonstrated that atrial structural remodeling plays a crucial role in the occurrence and maintenance of AF [109]. Furthermore, atrial structural remodeling (characterized by changes in both the size and shape of the left atrium) can be caused by AF itself, which, in turn, results in further progression of AF, a phenomenon referred to as ‘AF begets AF’ [110,111]. Left atrial enlargement has been an extensively used marker of atrial remodeling, though the left atrium is unsymmetrical, and left atrial dilatation frequently occurs not uniformly in all directions [112]. In the present AF family, significant atrial remodeling as reflected by the enlarged left atrial diameter was observed in the eldest living family member (II-2 from Family AF-01). However, significant atrial remodeling was not observed in the other living family members, which might be explained at least in part by the early echocardiographic detection at the initial diagnosis of AF. Given that left atrial changes represent an advanced stage of AF [111], long-term follow-up with close echocardiographic surveillance will probably discover significant atrial remodeling in the other living family members.

There are some limitations to the current investigation. First, the limited ethnic diversity of the study participants (from a single ethnicity, Han Chinese) and the small sample size may weaken the generalizability of the findings. Second, due to the intrinsic limitations of the WES analysis, we could not rule out the possibility that other genetic mutations might also have a role in the pathogenesis of AF. Specifically, regarding the WES analysis conducted, there are doubts about the robustness of the filtering process used to identify the *SOX4* mutation. The study’s reliance on rare, non-synonymous variations with a minor allele frequency of <0.1% might have missed other potential mutations or overemphasized the identified mutation. Third, the study’s findings that the *SOX4* mutations are novel and not found in databases like gnomAD and dbSNP are intriguing, but the study lacks broader validation. Fourthly, the mutation was found in all affected family members and none of the controls, yet the possibility of incomplete penetrance or variable expressivity could affect the interpretation of the results. Fifth, the study emphasizes the role of *SOX4* in AF, but the causative link between the mutation and AF is not definitively established, particularly without functional in vivo studies or replication in other ethnic populations. Sixth, we have demonstrated that SOX4 can transactivate the expression of GJA1/Cx43 and SCN5A, alone or in synergy with TBX5, but the complex interaction between SOX4 and the other different players, such as LMNA, PITX2, RyR2, and others for the development of AF, remains to be elucidated. Finally, there is a need for caution in making broad claims about the clinical implications of these findings without further validation.

## 5. Conclusions

In the current study, two novel *SOX4* loss-of-function mutations are identified to be accountable for idiopathic AF. The findings indicate *SOX4* as a new gene predisposing to AF and unravel a new molecular pathogenesis underlying AF, which provides a novel target for potential prophylactic and therapeutic intervention of AF in a subset of patients.

## Figures and Tables

**Table 1:** Baseline demographical and clinical characteristic profiles of the pedigree members with atrial fibrillation.

		Subject Information		Cardiac Phenotype	Electrocardiogram			Echocardiogram	
Identity (Family AF-01)	Sex	Age at Initial Diagnosis of AF (years)	Age at Recruitment (years)	AF (Clinical Classification)	Heart Rate (Beats/min)	QRS Interval (ms)	QTc (ms)	LAD (mm)	LVEF (%)
I-1	M	25	71 *	Permanent	NA	NA	NA	NA	NA
II-2	F	35	65	LSP	82	102	438	42	58
II-7	M	45	57	LSP	106	90	416	39	62
II-10	F	41	55	LSP	95	93	330	38	66
III-1	M	37	42	LSP	81	98	420	36	63
III-8	F	34	34	Persistent	84	88	432	33	67
IV-1	F	20	20	Paroxysmal	102	80	405	31	65

M, male; F, female; AF, atrial fibrillation; LAD, left atrial diameter; QTc, corrected QT interval; LVEF, left ventricular ejection fraction; LSP, long-standing persistent; NA, not available. * Age at death.

**Table 2:** Baseline demographic and clinical characteristic profiles of the cohort of 196 patients with idiopathic atrial fibrillation and the 278 control individuals.

Variable	Patient Group (*n* = 196)	Control Group (*n* = 238)	*p*-Value
Gender (female/male)	88/108	107/131	0.99000
Age (years)	56.93 ± 8.48	57.11 ± 9.27	0.8344
Family history of atrial fibrillation (%)	35 (18)	0 (0)	<0.0001 *
History of implanted pacemaker (%)	8 (4)	0 (0)	0.0032 *
History of ischemic stroke (%)	11 (6)	0 (0)	0.0003 *
Body mass index (kg/m^2^)	23.97 ± 2.52	24.12 ± 2.67	0.5506
Total cholesterol (mmol/L)	3.81 ± 0.73	3.78 ± 0.65	0.6511
Fasting blood glucose (mmol/L)	4.58 ± 0.64	4.60 ± 0.71	0.7603
Triglyceride (mmol/L)	1.49 ± 0.47	1.50 ± 0.51	0.8333
Diastolic blood pressure (mmHg)	82.97 ± 6.58	83.05 ± 7.13	0.9042
Systolic blood pressure (mmHg)	125.61 ± 9.73	126.23 ± 10.28	0.5222
Left atrial diameter (mm)	38.75 ± 7.61	36.04 ± 6.83	0.0001 *
Resting heart rate (beats/min)	76.02 ± 14.51	75.84 ± 10.33	0.8804
Left ventricular ejection fraction (%)	62.78 ± 7.09	63.04 ± 6.82	0.6980
History of smoking (%)	9 (5)	11 (5)	0.9882
History of alcohol consumption (%)	18 (9)	23 (10)	0.8648

* *p* < 0.05.

**Table 3:** Non-synonymous exon variants shared by all three members with atrial fibrillation (from Family AF-01) undergoing whole-exome sequencing assay.

Chr	Position (GRCh37)	Ref	Alt	Gene	Variation
1	67,185,057	A	G	*SGIP1*	NM_032291.4: c.1711A>G; p.(Asn571Asp)
1	210,267,717	C	T	*SYT14*	NM_001146261.4: c.628C>T; p.(Pro210Ser)
2	109,289,367	A	G	*LIMS1*	NM_001193485.3: c.562A>G; p.(Lys188Glu)
2	191,375,262	A	T	*NEMP2*	NM_001142645.2: c.955A>T; p.(Lys319*)
3	71,064,787	A	C	*FOXP1*	NM_032682.6: c.887A>C; p.(His296Pro)
4	139,980,574	G	A	*ELF2*	NM_201999.3: c.1311G>A; p.(Gly437Arg)
5	127,686,668	A	C	*FBN2*	NM_001999.4: c.2704A>C; p.(Ile902Leu)
6	21,594,976	C	T	*SOX4*	NM_003107.3: c.211C>T; p.(Gln71*)
7	64,292,159	C	A	*ZNF138*	NM_006524.4: c.461C>A; p.(Ser154*)
10	31,803,533	A	T	*ZEB1*	NM_001128128.3: c.639A>T; p.(Arg217Ser)
11	40,137,306	A	T	*LRRC4C*	NM_020929.3: c.537A>T; p.(Leu179Phe)
15	30,010,872	A	C	*TJP1*	NM_003257.5: c.3474A>C; p.(Glu1158Asp)
18	52,946,829	C	G	*TCF4*	NM_001083962.2: c.608C>G; p.(Ser203Cys)

Chr, chromosome; Alt, alteration; Ref, reference.

**Table 4:** Primers to amplify the complete coding exon of the human *SOX4* gene.

Coding Region	Forward Primer (5′→3′)	Reverse Primer (5′→3′)	Amplicon (bp)
Part 1	CTCTCTTTACCCACCTCCGC	GACCTTGTCTCCCTTCTCCC	643
Part 2	GCCCAGGAAGAAGGTGAAGT	GCGCCCTCCTCCTCGTACAG	603
Part 3	TGGCGGAGAAGAAGGTGAAG	TCGTCTGTCCTTTTCGTTTCT	647

## Data Availability

All the data related to this study are included in the manuscript.
